# Role of ARF6, Rab11 and External Hsp90 in the Trafficking and Recycling of Recombinant-Soluble *Neisseria meningitidis* Adhesin A (rNadA) in Human Epithelial Cells

**DOI:** 10.1371/journal.pone.0110047

**Published:** 2014-10-27

**Authors:** Giuseppe Bozza, Mirco Capitani, Paolo Montanari, Barbara Benucci, Marco Biancucci, Vincenzo Nardi-Dei, Elena Caproni, Riccardo Barrile, Benedetta Picciani, Silvana Savino, Beatrice Aricò, Rino Rappuoli, Mariagrazia Pizza, Alberto Luini, Michele Sallese, Marcello Merola

**Affiliations:** 1 Novartis Vaccines, Siena, Italy; 2 Unit of Genomic Approaches to Membrane Traffic, Fondazione Mario Negri Sud, S. Maria Imbaro (CH), Italy; 3 Institute of Protein Biochemistry, CNR, Naples, Italy; 4 Department of Biology, University of Naples “Federico II”, Naples, Italy; Cambridge University, United Kingdom

## Abstract

*Neisseria meningitidis* adhesin A (NadA) is a meningococcus surface protein thought to assist in the adhesion of the bacterium to host cells. We have previously shown that NadA also promotes bacterial internalization in a heterologous expression system. Here we have used the soluble recombinant NadA (rNadA) lacking the membrane anchor region to characterize its internalization route in Chang epithelial cells. Added to the culture medium, rNadA internalizes through a PI3K-dependent endocytosis process not mediated by the canonical clathrin or caveolin scaffolds, but instead follows an ARF6-regulated recycling pathway previously described for MHC-I. The intracellular pool of rNadA reaches a steady state level within one hour of incubation and colocalizes in endocytic vesicles with MHC-I and with the extracellularly labeled chaperone Hsp90. Treatment with membrane permeated and impermeable Hsp90 inhibitors 17-AAG and FITC-GA respectively, lead to intracellular accumulation of rNadA, strongly suggesting that the extracellular secreted pool of the chaperone is involved in rNadA intracellular trafficking. A significant number of intracellular vesicles containing rNadA recruit Rab11, a small GTPase associated to recycling endosomes, but do not contain transferrin receptor (TfR). Interestingly, cell treatment with Hsp90 inhibitors, including the membrane-impermeable FITC-GA, abolished Rab11-rNadA colocalization but do not interfere with Rab11-TfR colocalization. Collectively, these results are consistent with a model whereby rNadA internalizes into human epithelial cells hijacking the recycling endosome pathway and recycle back to the surface of the cell via an ARF6-dependent, Rab11 associated and Hsp90-regulated mechanism. The present study addresses for the first time a meningoccoccal adhesin mechanism of endocytosis and suggests a possible entry pathway engaged by *N. meningitidis* in primary infection of human epithelial cells.

## Introduction

*Neisseria meningitidis* (meningococcus) is a Gram-negative diplococcus that causes severe invasive disease and represents one of the most devastating bacterial infections. Although fatal if not treated on time, meningococcus invasion appears to be more an undesirable event of a usually commensal bacterium, probably due to a combination of host susceptibility and strain specific propensity to invasiveness [1], [2]. The pathophysiology of the bacterium *N. meningitidis* is a process that requires several steps: penetration of the epithelial or mucosal barrier, reaching and surviving the bloodstream, crossing the blood-brain barrier, and eventually causing meningitis through extracellular proliferation [3]. Epithelia are the primary target of bacterial colonization, an event basically asymptomatic and common to both non-virulent and virulent strains. Disease is a rare event compared to the extent of meningococcal nasopharynx colonization. [2], [4], [5]. Experimental data support attachment of the bacterium to nonciliated cells of the respiratory epithelium [6] and transcellular route of passage through this barrier [6]–[9]. A recent report shows that bacterial capsule and type 4 pili are important for epithelial cell transcytosis [9] but host and pathogen players involved in this process are far from being defined.

*Neisseria meningitidis* adhesin A (NadA) was identified through genome wide analysis and afterward proved to be expressed on the bacterial surface [10]–[12]. NadA is a trimeric outer membrane protein whose gene is present in three out of four known hypervirulent lineages of serogroup B strains [13], [14]. NadA is classified as a trimeric autotransporter adhesion (TAA), a family of outer membrane adhesin, which are present only in Gram negative bacteria. TAAs differ from classical autotrasporter protein by the homotrimeric structure hanging on the bacterial surface and the Type Vc secretion system [10], [11], [15]–[17]. Like other TAA proteins, NadA has a common modular organization, composed of i) a conserved C-terminal membrane anchor through which the protein is translocated to the cell surface, ii) a long central alpha helical domain (stalk) with high propensity to form coiled-coil structures, iii) an N-terminal globular head that has been associated with binding to specific cellular receptors and iiii) a cleavable signal sequence [18], [19]. Generally, TAAs (previously classified as Oligomeric coiled-coil Adhesins (Oca) family [20]) mediate bacterial interaction with host cells or extracellular matrix (ECM) proteins or induce invasion into target cells [21]–[29]. The specificity of the biological function is thought to reside in the head, while the stalk is thought to ensure adequate exposure of the head from the outer membrane [19].

In previous reports, we have used a NadA heterologous expression system to demonstrate the invasive ability acquired by an *Escherichia coli* strain exposing surface NadA. Our data support the role of NadA in the uptake of bacteria by Chang cells, a human epithelial cell line [10], [30]. A recombinant NadA (rNadA), expressed in *E. coli* and purified in a soluble form in absence of the anchor (translocator) domain, preserves its immunogenic properties and is included in a multicomponent vaccine against meningococcus B (Bexsero) [31], [32]. A peculiar feature of rNadA, perhaps unique among all members of the TAA family, is the ability to preserve a stable trimeric structure in solution [10], [13], [33]. This recombinant soluble homo-trimer still binds eukaryotic cells [10], [13], [33]–[35]. The gain-of-function phenotype acquired by heterologous bacteria expressing NadA and the conserved binding characteristics shown by the recombinant protein provide an opportunity to dissect the function of this adhesin in host-pathogen interaction(s).

When expressed on the surface of *E. coli*, NadA promotes bacterial internalization [10], by an undefined mechanism. Eukaryotic cells internalize a variety of extracellular substances as well as plasma membrane proteins via a remarkable diversity of endocytic pathways generally classified as clathrin-dependent or independent according to the involvement of clathrin [36]–[38]. Endocytic vesicles converge into early endosomes (EEs), the main sorting compartment from which most of the cargos are rapidly recycled back to the plasmamembrane (PM), while some are sorted into late endosomes and destined either to lysosomes or to the trans Golgi network (TGN) [39], [40]. While lysosome targeting is achieved by clathrin-coated endosomes, mechanisms of clathrin-independent endocytosis have been identified more recently and are emerging as complex, multi-pattern processes of internalization still poorly characterized [41], [42]. In particular, ADP ribosylation Factor 6 (ARF6) has been involved in internalization and sorting of cargos targeted to recycling endosome [43]. Initially described for the major histocompatibility complex (MHC) class I trafficking [44], the list of membrane proteins requiring this factor for internalization and recycling is growing [42], [43]. While ARFs coordinate vesicle formation [45], key regulators of endosome trafficking include the Rabs, a family of small GTPase proteins associated to all kind of vesicles [46]. The more than 60 Rabs found in eukaryotic cells communicate with each other through recruitment of effectors that regulate the correct destination of vesicles [39], [47].

In our attempt to identify eukaryotic cell proteins involved in the NadA-mediated interaction of bacteria, we found that such processes are sensitive to inhibition of Hsp90 activity, and the host chaperone itself was found to bind NadA *in vitro* [30]. Hsp90 is a ubiquitous molecular chaperone crucial for maintenance of cellular homeostasis [48]. The Hsp90 family of chaperones includes organelle specialized isoforms and two cytoplasm/nucleus localized factors, the inducible Hsp90α and the constitutive Hsp90β. More recently, several lines of evidence suggest that Hsp90α is also secreted by normal cells in response to stress or insults, and by several tumor cell lines (reviewed in [49]). The major functions of this extracellular pool have been identified as protective response to several kinds of stress, assistance to antigen presentation and promoting tissue invasion by cancer cells [49]–[52]. Results from our previous study also indicated a protective role of Hsp90 toward NadA mediated invasion, although we did not differentiate the possible role of extracellular and intracellular pools of the chaperone [30].

In this report we have used the soluble recombinant NadA_Δ351-405_ (rNadA) and demonstrated that it was able to internalize upon binding to the Chang human epithelial cell line. Evidence is provided on the stability of the rNadA trimer at 37°C and on the temperature dependence of NadA binding to Chang cells. In our analysis of the internalization pathway exploited by NadA, we found that the process triggered by NadA was clathrin independent. As reported for MHC class I internalization and recycling to cell surface, ARF6-dependence and recruitment of Rab 11 on rNadA vesicles suggested that the adhesin is sorted in a recycling endosomes pathway. The effect of two Hsp90 inhibitors on the internalization and recycling of the adhesin indicated that extracellular Hsp90 is involved in the trafficking of rNadA.

## Materials and Methods

### Cell culture and transfection

Chang epithelial cells (Wong-Kilbourne derivative, clone 1-5c-4, human conjunctiva, ATCC CCL-20.2) were grown in Dulbecco's Modified Eagle Medium (DMEM) supplemented with 15 mM glutamine, 100 U/ml penicillin, 100 µg/ml streptomycin, 10% FCS and maintained at 37°C in a controlled humidified atmosphere containing 5% CO_2_. Transfection of Chang cells was performed using Hiperfect (Qiagen), jetPEI (Polyplus transfection) or lipofectamine (Invitrogen) according the manufacturer's instructions.

### Antibodies, plasmid vectors and reagents

Anti-MHC-I (Biolegends); anti-EEA1 (Novus Biologicals); anti-ARF6 (Santa Cruz Biotechnology); anti-Rab5 (BD transduction Laboratories); anti clathrin (Sigma); anti-M6PR kindly provided by J. Gruenberg University of Geneva, Switzerland; anti-HSP90 (SPA-830, Stressgene); Alexa-488-conjugated Phalloidin, Cy3-conjugated transferrin, Alexa488- and 543-conjugated secondary antibodies (Invitrogen); Alexa488-conjugated anti-MHC-I (Santa cruz Biotechnology). Antibodies against rNadA were previously described in [53]. Alexa-488-conjugated and Alexa-633 conjugated rNadA were obtained by labelling with Alexa Fluor Microscale Protein Labeling Kit (Molecular Probes-Invitrogen) following manufacturer instructions. cDNA expressing the wild type and ARF6-Q67L mutant were kindly provided by J. Donaldson (to M.S. and A.L.), National Institutes of Health, Bethesda, USA. 17-AAG (17-N-allylamino-17-demethoxygeldanamycin) and Wortmannin were from Sigma. FITC-GA was synthesized by labogen (Milan - Italy). The myc-AP180C plasmid containing the C-terminal portion of the AP180 gene fused in frame with N-terminal myc tag sequence was generated as described elsewhere [54]. Generation of plasmids coding for C-terminal EGFP fusion Rabs was performed as described previously [55].

### Binding of rNadA to human Chang epithelial cells

Chang cells were non-enzymatically detached from the support by using cell dissociation solution (Sigma), harvested and resuspended in DMEM medium supplemented with 1% FBS.

Then 3×10^5^ cells were mixed with recombinant rNadA (200 µg/ml) for 30 minutes at four different temperatures (RT, 4°C, 30°C and 37°C). After two washes with 1% FBS in phosphate-buffered saline, cells were incubated with the mouse monoclonal anti-NadA antibody 9F11 (1.25 µg/ml to 160 µg/ml) or IgG mouse isotype control (Sigma) for 1 hr at 4°C. Samples were washed twice and then incubated for 30 minutes at 4°C with Allophycocyanin-conjugated goat F(ab)_2_ antibody to mouse IgG (1∶100, Jackson ImmunoResearch) and finally the cells were analyzed with the FACS-Scan flow cytometer Canto II. The mean fluorescence intensity for each sample was calculated.

To analyze the time dependence of NadA binding to Chang, 3×10^5^ cells were incubated with recombinant rNadA (200 µg/ml) at 37°C for different period of time (1 minute to 2 hours), washed and incubated with the mouse monoclonal anti-NadA antibody 9F11 (20 µg/ml) or IgG mouse isotype control for 1 hr at 4°C. FACS analysis was performed as described above.

### Size Esclusion-HPLC–multiangle laser light-scattering (MALLS) analysis

Four identical aliquots (100 µl) of rNadA (1,7 mg/ml in 10 mM NaH_2_P0_4_ 150 mM NaCl pH 7.2) were heated at 37°C for 30 min, 1, 2 and 4 hrs respectively. For each time point, sample was loaded on an analytical size exclusion TSK Super SW3000 (4.6×300-mm column of 4 µm and 300 Å) with a separation range on globular proteins from 10 to 500 kDa (Tosoh Bioscience, Tokyo, Japan). Samples were eluted isocratically in 0.1 M NaH_2_P0_4_, 0,1 M NaHSO_4_ buffer at pH 7.0. MALLS analyses were performed in real time using a multi-angle light-scattering detector Dawn TREOS (Wyatt Corp., Santa Barbara, CA, USA), with an incident laser of 658 nm; intensity of the scattered light was measured at 3 angles simultaneously. Data elaboration was performed using Astra V software (Wyatt). Zimm formalism was used to determine the weight-average molecular mass (MW) in daltons and polydispersity index (MW/Mn) for each oligomer present in solution.

### Full length trimeric NadA purification on size exclusion chromatography

A 300 µl aliquot of rNadA (1,7 mg/ml Buffer 10 mM NaH_2_P0_4_ 150 mM NaCl pH 7.2) was heated at 37°C for 4 hours. At end of this time period, sample was loaded on the gel filtration column. Size exclusion was performed using a Superdex 75 10–300 GL (GE-Healtcare) and elution was performed isocratically in 0.1 M NaH_2_P0_4_ pH 7.2, 0.15 M NaCl. Protein containing fractions were identified by OD at 280 nm and collected.

### Immunofluorescence staining and confocal microscopy

Chang cells were cultured on coverslips to reach 70–80% of confluence, washed three times in medium without serum and then incubated with rNadA (200 µg/ml) in the same medium supplemented with 1% FCS. Incubation was performed at 37°C for the indicated times. Cells were then fixed in paraformaldehyde for 8–10 min at 37°C, permeabilized and incubated with primary antibodies for 1 hr at room temperature. Afterwards, cells were washed three times with PBS, incubated with fluorophore-conjugated secondary antibody for 30–45 min at room temperature, washed again three times in PBS and mounted on slides.

*In vivo* staining of MHC-I was performed as follows: cells were washed three times in medium without serum and incubated with a mouse monoclonal antibody against MHC-I (10–30 µg/ml) for 1 hr at 4°C. Afterwards, cells were washed 3 times and incubated with recombinant rNadA (200 µg/ml) for 1 hr at 37°C in a medium supplemented with 1% FCS, then fixed and permeabilized. rNadA was stained following the standard procedure while MHC-I was revealed using a fluorescence-conjugated secondary antibody directed against the mouse monoclonal primary antibody.

*In vivo* staining of HSP90 was performed as follows: cells were washed three times in medium without serum and incubated with a rabbit polyclonal HSP90 antibody (50 µg/ml) for 2–4 hrs at 4°C. Afterward, cells were washed 3 times and incubated with recombinant rNadA (200 µg/ml) for 1 h at 37°C in a medium supplemented with 1% FCS, then fixed and permeabilized. rNadA was stained following the standard procedure while HSP90 was detected using a Alexa543-conjugated secondary antibody directed against the rabbit polyclonal primary antibody.

Samples were analyzed by confocal microscopy (LSM 510, Zeiss) using a 60× oil-immersion objective, maintaining the pinhole of the objective at 1 airy unit. Images were scanned using an Argon 488 laser, a HeNe 543 laser and a HeNe 633 laser, under non-saturating conditions (pixel fluorescence below 255 arbitrary units).

The colocalization analysis and the quantification of immunofluorescence (IF) intensity of rNadA in the cells was performed with LSM510-3.2 software (Zeiss). To assess the colocalization we removed the background immunofluorescence by adjusting the threshold levels and used the histo and colocalization functions of the above software. This software provides two colocalization coefficients that ranges from 0 (no colocalization) to 1 (complete colocalization). The colocalization coefficients indicate the amount of pixels of the channel A that colocalizes with pixels from channel B and viceversa. Finally, we expressed the colocalization extent as a percentage over the total immunofluorescence per channel. The immunofluorescence (IF) intensity was calculated as total immunofluorescence of rNadA in the cell divided by the area of the cell and expressed as arbitrary units (A.U.).

### rNadA uptake in the presence of Hsp90 inhibitors

Internalization was performed by adding rNadA to the culture medium at a final concentration of 200 µg/ml and incubating at 37°C for the indicated period of time. Chang cells grown at about 50% confluence were pre-treated overnight with 0.5 µM 17-AAG and the then incubated with recombinant NadA (200 µg/ml) at 37°C for 1, 4 or 16 hrs in presence of the same concentration of 17-AAG. When 10 µM 17-AAG or FITC-GA were used, cells were grown to 70–80% confluence and pretreated for 1 hr with the inhibitors before adding rNadA (200 µg/ml) and subsequently incubated at 37°C for 1,4 or 16 hrs in the continued presence of the inhibitors. To temporarily permeabilize cells, growth medium was substituted with 0.01% saponin in PBS for 30 seconds at room temperature. Cells were then washed 3 times with PBS before adding fresh medium and incubate at 37°C for the indicated time. Afterwards, cells were fixed and labeled as detailed above.

### AKT detection in Chang cell lysates

Chang cells were seeded on 24-well tissue culture plates (6–8×10^4^ cells per well) and incubated at 37°C overnight. The following day, cells were treated with HSP90 inhibitors as described above. Total cell extracts were prepared in RIPA buffer (Sigma) supplemented with complete protease inhibitor (Roche).

Equal amounts of proteins were prepared in NuPAGE SDS Buffer under reducing conditions and separated on NuPAGE polyacrylamide gels. Proteins were transferred to nitrocellulose membranes for Western blot analysis. Membranes were then blocked with PBS containing 0.1% Tween 20 (PBST)+10% dried skim milk at room temperature for 1 h. After extensive washings in PBST, proteins bound on nitrocellulose membranes were detected with specific anti-Akt rabbit antibody (Cell Signaling Technology) followed by HRP-conjugated goat anti-rabbit secondary antibody.

### Time-lapse Microscopy

Chang cells at 70–80% of confluence on glass-bottom Petri dishes (MatTek Corporation-USA) were incubated overnight at 37°C. The day after, cells were incubated with Alexa488-conjugated anti-MHC-I antibody and 2.5 µg/ml Alexa633-conjugated rNadA, or 5 µg/ml of Cy3-conjugated transferrin and 2.5 µg/ml Alexa-488-conjugated rNadA, or pretreated overnight with 0.5 µM of 17AAG and then with Alexa-488-conjugated rNadA for 60 min at 37°C. Cells were washes three times and supplemented with the medium buffered with 20 mM Hepes pH 7.2. The Petri dish was then placed on a stage of LSM-510 confocal microscope (Zeiss, Germany) equipped with a thermoregulation device at 37°C. Images were acquired every 2 (for MHC-I and NadA; movie S1) or 4 seconds (for transferrin and rNadA, movie S2; rNadA in 17AAG treated or untreated cells, movie S3 and S4 respectively) at 25% laser power intensity.

## Results

### Purified rNadA binds Chang cells in a time and temperature dependent manner

In previous reports, rNadA binds Chang epithelial cells more than other human cell lines [10]. A temperature dependence has also been suggested, as binding to human monocytes/macrophages was detectable at 37°C but not at 0°C [35]. We sought to investigate the temperature dependence and kinetics of NadA binding to Chang epithelial cells. Non permeabilized Chang cells were incubated with rNadA and, after washing, the bound fraction was revealed by FACS analysis with anti-NadA antibody.

**Figure 1:**
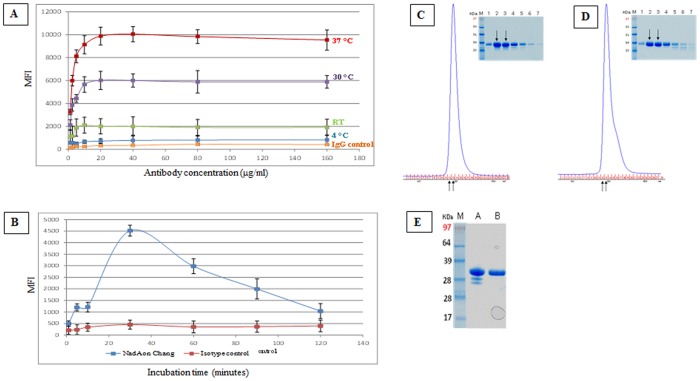
Purified rNadA binds Chang cells in a time- and temperature dependent manner. **A and B**. Chang cells were incubated with 200 µg/ml rNadA and then washed with PBS – 1% FBS. Binding was detected using anti-NadA 9F11 mouse mAb and Allophycocyanin-conjugated goat anti-mouse secondary antibody. Analysis was performed with Canto II instrument, and mean fluorescence intensity is reported (MFI). ***A***: Binding of rNadA to cells for 30 minutes at the indicated temperatures, and using varying concentrations of the primary antibody ***B***: Binding of rNadA was performed at 37°C for a period of time ranging from 1 minute to 2 hours as indicated. ***C***: SE-HPLC profile of rNadA at room temperature. ***D***: SE-HPLC profile of rNadA after heating period of 4 hrs at 37°C. ***E***: SDS-Page of collected fractions. Black arrows in **D** indicate the fractions pooled.

At first this analysis was performed after 30 minutes of incubation at different temperatures. The result is shown in figure 1A. The rNadA binding to Chang cells was temperature dependent, giving the highest value at 37°C while dropping to about 60% at 30°C (figure 1A). Lowering the incubation temperatures at 20°C caused a decrease of the binding ability at less than 20% of the value obtained at 37°C. However, even at 4°C the amount of cell-bound rNadA was measurably higher than the background (independent IgG used as control) Setting incubation temperature at 37°C, we then analyzed the kinetics of rNadA binding to Chang cells (figure 1B). The maximum level of surface bound rNadA was observed at 30 minutes of incubation and decreased at longer incubation times. The time-dependent decrease of surface localized rNadA was obtained with various monoclonal and polyclonal antibodies used for detection (data not shown).

To exclude a destabilization of the trimeric rNadA structure at 37°C over time, we incubated the recombinant adhesin for 30 min, 1, 2 and 4 hrs and analyzed the products by analytical size exclusion chromatography and MALLS (Multi Angle Laser Light Scattering). The results of such analysis, presented in figure S1 and table S1, showed that the native trimer is the predominant species (about 95%) although the rNadA preparation include C-terminus deleted forms previously characterized [33]. The 4 hour treatment at 37°C completely removed these truncated species and the full length homogenous trimeric population can be purified as shown in figure 1, panels C–E.

The size exclusion chromatography analysis of the sample before and after thermal treatment is shown in figure 1C and 1D, respectively. The SDS-PAGE of the eluted samples confirmed that all contaminants were removed from the fractions containing the majority of the trimeric rNadA following 4 hour incubation at 37°C (fractions 12 and 13 of figure 1C and 1D). The purified full length trimeric rNadA eluted in peak II (lane B of figure 1E) was used at least once in all our experiments and challenged with the crude rNadA preparations. Both samples gave identical results. Thus, trimers of rNadA form a stable structure in solution which is resistant to the temperature conditions used in our studies.

### rNadA internalization into Chang epithelial cells

**Figure 2:**
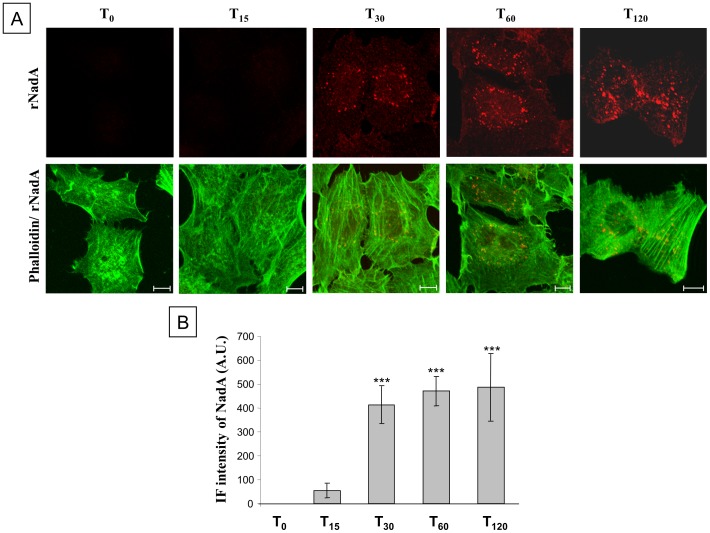
Time course of rNadA internalization. ***A:*** Chang cells were incubated with 200 µg/ml rNadA at 37°C for 0–120 min and then fixed, permeabilized and double stained for rNadA (upper panels; red) and Alexa488-conjugated phalloidin (green). Merged images of rNadA and phalloidin are shown in the lower panel. Scale bar is 10 µm. ***B:*** Quantification of IF intensity of rNadA in the experiment illustrated in panel A. Data are mean ± s.e.m representative of two independent experiments, each assessing 10–15 cells, and expressed as Arbitrary Units (A.U.). ***p<0.001, compared to T_0_ treated cells (t-test).

Having excluded that the time-dependent reduction of rNadA binding to Chang cells was caused by changes in the trimeric structure or stability of the protein, we investigated a possible internalization process by confocal immunofluorescence microscopy (figure 2).

Following 15 minutes of incubation with rNadA, cells showed minor amounts of detectable rNadA apparently distributed on the cell surface (figure 2). Upon 30 minutes of incubation, rNadA appeared mostly as dot-like structures. At a longer time of incubation the fluorescence of the dot-like structures slightly increased (figure 2A and B). Hypothesizing that the dot-like structures are indicative of rNadA internalization, this phenomenon could explain, at least partially, the observed loss of rNadA fluorescence observed by FACS analysis at prolonged incubation times. However, the confocal microscopy also indicated that the intracellular pool of rNadA reaches a steady state level at about 30–60 minutes incubation time, possibly because it recycles towards the cell exterior or because of intracellular degradation.

### Phosphoinositide 3-kinase (PI3K) control of rNadA internalization

Src phosphorylation of several components of the clathrin dependent endocytosis, including cortactin, arrestin, dynamin and clathrin itself, is essential for internalization [56]. In addition, Src activity is also required for the alternative caveolar endocytosis [57]. Protein kinase C is involved in the endocytosis of several G protein coupled receptor through clathrin or caveolae pathways [58], [59]. The phosphoinositide 3-kinase (PI3K) is important in the early steps of endocytosis and and in the regulation of fusion and maturation dynamics of endocytic vesicles [60]. Here we used inhibitors of the different kinases to better understand the internalization pathway(s) exploited by rNadA.

**Figure 3:**
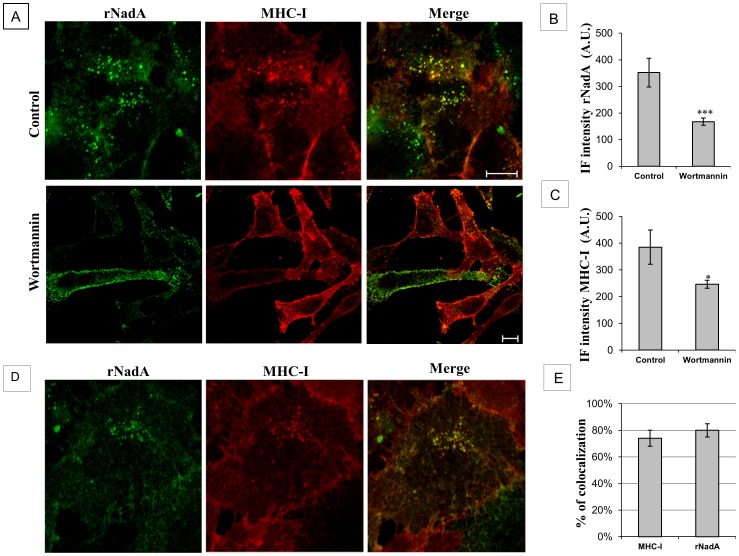
PI3K inhibition impairs rNadA internalization and colocalization of rNadA with MHC-I. ***A:*** Chang cells were treated with either vehicle (upper panel) or 100 nM Wortmannin (lower panel) for 1 h at 37°C, and then incubated with 200 µg/ml rNadA for an additional hour at 37°C in presence of the inhibitor or vehicle. Cells were then fixed, permeabilized and double stained for rNadA (green) and MHC-1 (red). Merged images are also shown. Scale bar 10 µm. ***B:*** Quantification of IF intensity of rNadA in the experiment illustrated in panel A. Data are mean ± s.e.m representative of two independent experiments, each assessing 20–25 cells, and expressed as Arbitrary Units (A.U.). ***p<0.001, compared to control cells (t-test). ***C:*** Quantification of IF intensity of MHC-I in the experiment illustrated in panel A. Data are mean ± s.e.m representative of two independent experiments, each assessing 20–25 cells, and expressed as Arbitrary Units (A.U.). *p<0.05, compared to control cells (t-test). ***D:*** Chang cells were treated with anti-MHC-I antibody for 1 h at 4°C and then incubated at 37°C for 1 h with 200 µg/ml rNadA. Cells were fixed and double stained for rNadA (green) and MHC-1 (red). Scale bar is 10 µm. ***E:*** Quantification of MHC-I and rNadA colocalization. MHC-I column indicate the percentage of MHC-I immunofluorescent pixels colocalizing with rNadA immunofluorescent pixels. Conversely, rNadA column indicate the percentage of rNadA immunofluorescent pixels colocalizing with MHC-I immunofluorescent pixels. Data are mean ± s.e.m representative of two independent experiments, each assessing 20–25 cells.

Chang cells were incubated with or without kinase inhibitorsfor 1 hour with vehicle, 100 nM wortmannin (PI3K inhibitor more active on class I enzymes), 100 µM genistein (wide spectrum tyrosine kinase inhibitor known to prevent the caveolae mediated endocytosis [61]), 10 µM Bisindolylmaleimide I (BSMI, a general PKC inhibitor), and 10 µM SU6656 (Src family kinase inhibitor) and then for an additional hour with rNadA. The internalization of rNadA was unaffected by the action of the tyrosine kinase inhibitors (data not shown) whereas wortmannin treatment reduced the internalization of rNadA (figure 3A and B). The cellular localization of MHC-I was unaffected by exposure to rNadA, genistein, BSMI and SU6656 (not shown), whereas wortmannin shifted the localization of MHC-I towards the PM fraction (figure 3A and C).

In these experiments, rNadA showed a certain extent of endosomal colocalization with MHC class I and we decided to further explore this observation. Antibody labelling of MHC-I following cell permeabilization would show the total pool of MHC-I (PM plus endosomally localized MHC-I). In order to visualize only the recently internalized MHC-I we decided to pre-label the PM-localized MHC-I. Chang cells were treated with an antibody against MHC-I for 1 hr at 4°C. This incubation allowed binding of the antibody to MHC-I at a temperature that prevents MHC-I internalization. The cells were then treated with rNadA for 60 minutes at 37°C, a temperature at which internalization is restored. As shown in figure 3D and E, rNadA appeared to colocalize with MHC-I in endosomes.

### Confocal microscopy analysis of rNadA internalization pathway

In an attempt to elucidate the cellular entry mechanism of rNadA, we performed a confocal microscopy analysis in search of rNadA colocalization with different endocytic markers belonging to the clathrin-dependent and -independent pathways.

**Figure 4:**
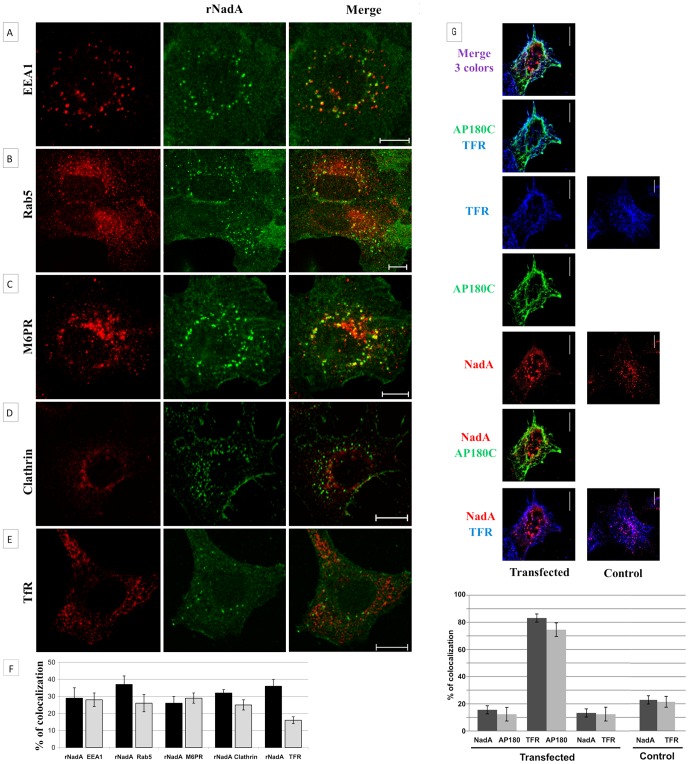
Colocalization of rNadA with endosome markers. ***A:*** Chang cells were incubated with 200 µg/ml rNadA for 1 h at 37°C, then fixed, permeabilized and double stained for rNadA (green) and EEA1 *(* ***A*** *)*, Rab5 *(* ***B*** *)*, M6PR *(* ***C****)*, Clathrin *(* ***D*** *)* and TfR *(* ***E*** *)* (red). Merged images of the red and green signals are also shown. Images are representative of two independent experiments. Scale bar is 10 µm. ***F:*** Quantification of rNadA colocalization with endosome markers. The black columns indicate the percentage of rNadA immunofluorescent pixels colocalizing with the endosomal marker (EEA1, RAb5, M6PR, Clathrin, TfR as indicated) immunofluorescent pixels. Viceversa the grey columns indicate the percentage of the endosomal marker immunofluorescent pixels colocalizing with rNadA immunofluorescent pixels. Data are mean ± s.e.m representative of two independent experiments, each assessing 20–25 cells. ***G:*** Chang cells were transfected with an EGFR-AP180C expressing plasmid or with empty vector and incubated 24 hours at 37°C to recover. Cells were then incubated with rNadA, fixed and stained as indicated in point A. EGFR-AP190C is shown in green, rNadA in red and TfR in blu. Quantification was performed as indicated in point F.

Chang cells were incubated with rNadA for 60 minutes at 37°C to allow internalization of the adhesin and then stained for rNadA and the endocytic markers, early endosome antigen 1 (EEA1), small GTPase Rab5, M6PR. As shown in figure 4A–C and F, rNadA showed a weak colocalization with early endosomal markers (EEA1, Rab5), as well as with the endo-lysosomal marker M6PR. We also analyzed the colocalization of internalized rNadA with clathrin itself and the transferrin receptor, a prototype cargo of the clathrin-dependent pathway. Remarkably, rNadA containing carriers did not associate with the scaffold protein clathrin, and the colocalization with transferrin receptor was negligible (figure 4D–F). As a control, we showed that transferrin receptor colocalized with clathrin in Chang cells (figure S2). Since these data suggested that the main rNadA internalization pathway does not rely on the clathrin- pathway, we sought to verify this aspect by the analysis of rNadA internalization in presence of an inhibitor of clathrin vesicles formation. The AP180 protein is a crucial adaptor involved in the early stage of chlatrin-dependent endocytosis [54].Expression of the C-terminus moiety of this adaptor, known as AP180C, inhibits transferrin and EGF receptors clathrin-dependent endocytosis by preventing coat formation and leading to clathrin redistribution [54], [62]. AP180C transfected Chang cells were incubated with rNadA for 60 minutes at 37°C before to be fixed and treated for confocal analysis. As control, we followed TfR internalization both in absence and presence of the truncated AP180. Results are shown in figure 4G and quantification of rNadA, TfR and AP180C colocalization reported in graph. As expected, TfR internalization was severely impaired by AP180C expression while the intracellular vesicular pattern of rNadA was not affected (figure 4G). Furthermore, the absence of rNadA colocalization with either TfR or AP180C confirmed that the two proteins follow different internalization pathways.

To further explore the internalization pathway used by rNadA, we analyzed the presence of rNadA in the lysosomal compartment. rNadA showed a very minor colocalization with the lysosomal marker LAMP1 upon 60 minute of incubation as well as at longer incubation times up to 24 hours (not shown). This observation is consistent with a small presence of the adhesin in the early endosomal compartment and indicates that internalized rNadA is not directed towards the degradative lysosomal pathway.

**Figure 5:**
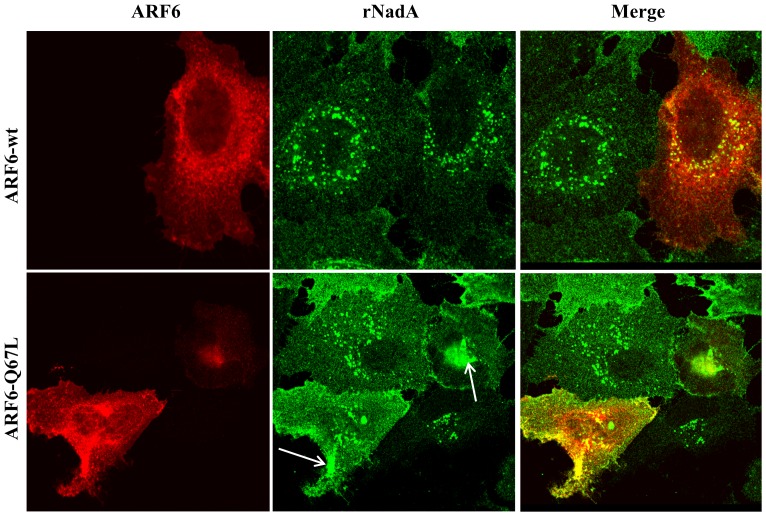
rNadA intracellular distribution is affected by ARF6. Chang cells were transfected in order to overexpress wild type ARF6 (ARF6-wt) or dominant-negative ARF6 (ARF6-Q67L) and then incubated overnight with 200 µg/ml rNadA at 37°C. Afterwards, cells were fixed, permeabilized and double stained for rNadA (green) and ARF6 (red). Merged images are also shown. Scale bar 10 µm. Images are representative of two independent experiments.

Finally, we analyzed the involvement of the clathrin-independent ARF6-regulated pathway. To verify whether endosomal sorting of internalized rNadA involves the small GTPase ARF6 [60] we monitored the internalization/recycling of rNadA in Chang cells expressing wild-type, and GTP-locked ARF6 (ARF6-Q67L). Previous studies demonstrated that ARF6-Q67L transfection leads to the intracellular accumulation of MHC-I [60]. Indeed, when mutant cells overexpressing ARF6-Q67L were incubated with rNadA overnight there was an accumulation of intracellular rNadA, while in cells overexpressing wild-type ARF6 the classical spotted distribution of rNadA was observed (figure 5).

In conclusion, this indicates that the rNadA endosomal trafficking involves the clathrin independent ARF6-regulated pathway. The carriers generated along this pathway can fuse with the early endosome, or take a more direct route towards the PM [60], [63], [64]. Indeed, our data suggest that rNadA may use a more direct recycling pathway without involvement of the early endosomal compartment. This hypothesis would be consistent with the negligible colocalization with the lysosomal compartment [64].

### rNadA, MHC-I and transferrin internalization dynamics in live cells

**Figure 6:**
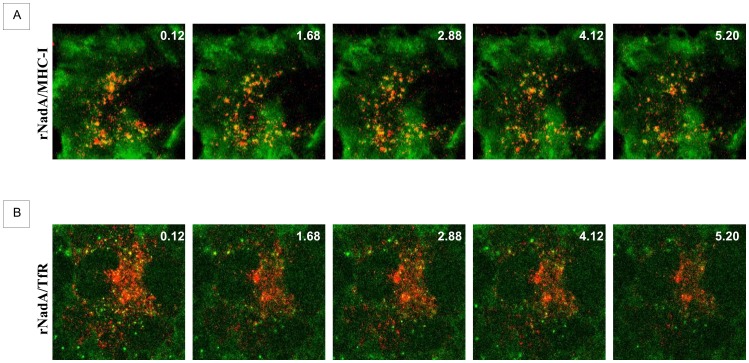
Time lapse rNadA internalization. ***A:*** Chang cells were incubated for 1 h with Alexa633-conjugated rNadA (shown in red) and Alexa488-conjugated anti-MHC-I antibody (green). Cells were then washed and live images were recorded every 2 seconds by confocal microscopy. Five frames from the Movie S1 are shown. Time (seconds) is indicated in the top right of each panel. ***B:*** Chang cells were incubated for 1 h with Alexa488-conjugated rNadA (green) and Cy3-conjugated transferrin (red). Cells were then washed and live images were recorded every 4 seconds by confocal microscope. Five frames from the Supplementary Movie S2 are shown. Time (seconds) is indicated in the top right of each panel.

In order to validate that rNadA uses the MHC-I internalization/recycling route we investigated their co-internalization in live cells. Chang cells were incubated for 1 hour with fluorescent Alexa633-conjugated rNadA and fluorescent Alexa488-conjugated anti-MHC-I antibody. Cells were rapidly washed with PBS and imaged for 5 minutes by confocal microscope. The movies showed the presence of rapidly moving carriers, containing both rNadA and MHC-I (figure 6A and movie S1). A minor fraction of carriers contained either rNadA or MHC-I. This could be due to the previously described dual targeting of surface MHC class I [65].

To cross-check that the rNadA internalization route is clathrin-independent, we tested the overlap between endocytosed rNadA and transferrin, a cargo internalized via the clathrin-dependent pathway. Chang cells were incubated for 1 hour with fluorescent Alexa488-conjugated rNadA and fluorescent Cy3-conjugated transferrin. Cells were rapidly washed with PBS and imaged as above. The movies showed the presence of rapidly moving rNadA or transferrin containing carriers indicating that the intracellular trafficking of rNadA is essentially different from that of transferrin (figure 6B and movie S2). As expected, only a minor fraction of carriers showed the presence of both rNadA and transferrin, perhaps as a result of a sorting/recycling compartment where the clathrin-dependent and independent pathways converge. Taken together, these observations confirm that the intracellular trafficking route of rNadA is clathrin independent and mostly overlaps with that of MHC-I.

### Functional involvement of intracellular and external Hsp90 in internalization and trafficking of rNadA

In our recent report [30], we identified Hsp90 as a cellular factor interfering with rNadA-mediated bacterial infection. Although Hsp90 is an intracellular chaperone, its presence in the extracellular space has also been reported [50]. Here, we sought to verify the possible relationship between extracellular Hsp90 and rNadA internalization.

**Figure 7:**
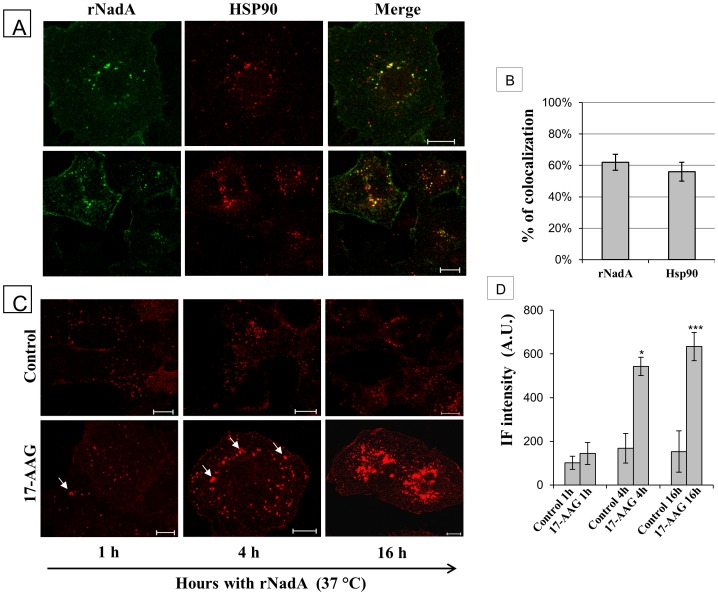
Colocalization of rNadA with HSP90. ***A:*** Chang cells, stained *in vivo* with a rabbit polyclonal anti-HSP90, were incubated with rNadA for 1 h at 37°C, then fixed, permeabilized and double stained for rNadA (green) and HSP90 (red). Panels are taken from two different experiments. Merged images are also shown. Scale bar 10 µm. ***B:*** Quantification of rNadA and Hsp90 colocalization. rNadA column indicate the percentage of rNadA immunofluorescent pixels colocalizing with HSP90 immunofluorescent pixels. Conversely, HSP90 columns indicate the percentage of HSP90 immunofluorescent pixels colocalizing with rNadA immunofluorescent pixels. Data are mean ± s.e.m representative of two independent experiments, each assessing 20–25 cells. ***C:*** Chang cells pre-treated overnight with either vehicle (upper panel) or 0.5 µM 17-AAG (lower panel), were incubated with 200 µg/ml rNadA for 1 h, 4 h and 16 hrs as indicated, then fixed, permeabilized and stained for rNadA (red). Intracellular accumulation of rNadA clusters are indicated by arrows. Scale bar 10 µm. ***D:*** Quantification of IF intensity of rNadA in the experiment illustrated in panel C. Data are mean ± s.e.m representative of two independent experiments, each assessing 20–25 cells, and expressed as Arbitrary Units (A.U.). ***p<0.001, compared to 1 h of 17-AAG treated cells (t-test). *p<0.05, compared to 1 h of 17-AAG treated cells (t-test).

To this end, we selectively labeled the external Hsp90 in live cells by incubating them with an antibody against Hsp90 for 1 hr at 4°C. This approach allows the binding of the antibody to Hsp90 present on the external side of the PM, while the low temperature prevents internalization. Excess unbound anti-hsp90 antibodies were removed and the cells were treated with rNadA for 60 minutes at 37°C. Finally, the cells were fixed and rNadA was labeled with the appropriate primary and secondary antibodies. The anti-Hsp90 antibodies were revealed using fluorescence-conjugated secondary antibodies. As shown in figure 7A, externally labelled Hsp90 accumulated intracellularly in compartments containing rNadA, strongly suggesting that Hsp90 present on the external side of the PM is internalized and colocalizes with the rNadA (figure 7B).

The functional role of Hsp90 in rNadA internalization was further investigated using the specific Hsp90 inhibitor 17-AAG. Chang cells pre-treated overnight with 0.5 µM of 17-AAG were then incubated with rNadA for 1, 4 and 16 hours. After 1 hour of rNadA incubation in 17-AAG-treated cells, clusters of rNadA appeared as dot-like structures as compared to control inhibitor-untreated cells (figure 7C, left panels and D). At longer times, clusters of rNadA-containing structures increased in size and number resulting in a remarkable accumulation of rNadA within the cells (figure 7C, central panels and D). At 16 hours the intracellular rNadA amount was roughly double in 17-AAG treated cells compared to control cells (figure 7C, right panels and D). Finally, we investigated the rNadA dynamics in 17-AAG treated cells. Chang cells were pre-treated overnight with 0.5 µM 17-AAG, incubated for 1 hour with fluorescent Alexa488-conjugated rNadA, rapidly washed with PBS and a movie recorded as above. In contrast to the rapidly moving rNadA containing carriers of the control cells, in 17-AAG treated cells, carriers containing rNadA appeared less mobile. In addition, their fusion to form larger structures or the emergence of small rNadA carriers from these aggregate appeared less frequent in 17-AAG than in controls (movie S3 and S4).

**Figure 8:**
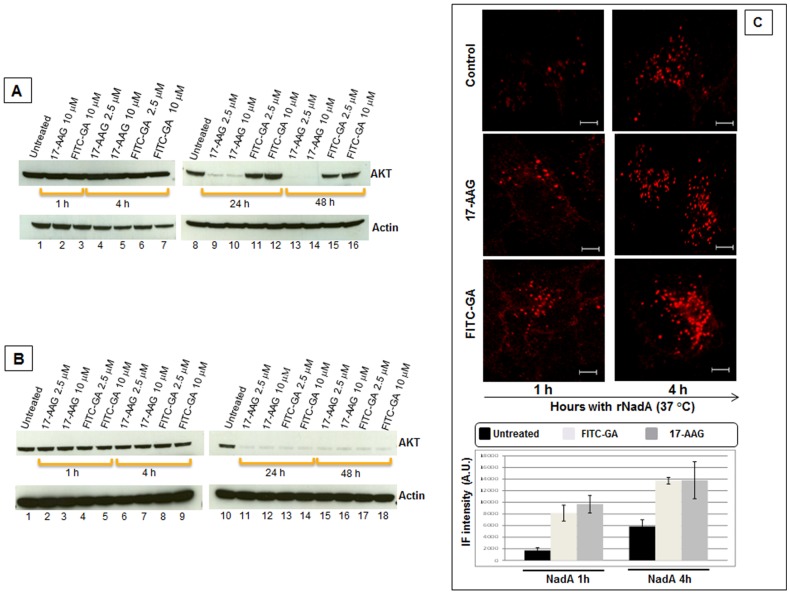
Inhibition of Hsp90 influences AKT degradation and intracellular accumulation of rNadA. ***A:*** Chang cells were incubated for 1, 4, 24 and 48 hours with either 17-AAG or FITC-GA at two different concentrations. At the end of each period cells were washed, trypsinized and RIPA-buffer total lysate prepared. Proteins from each extract were separated on NuSieve gel, blotted, and AKT or Actin (loading control) were detected using the respective primary antibodies. ***B:*** Chang cells were pre-treated with 0.01% saponin in PBS for 30 seconds at room temperature, washed three times with PBS and then handled as described in A. ***C:*** Chang cells were pre-treated for 1 hour with either vehicle (upper panels), 10 µM 17-AAG (middle panel) or 10 µM FITC-GA (lower panel), and then incubated with 200 µg/ml rNadA at 37°C for 1 (left panels) or 4 hours (right panels). Cells were then fixed, permeabilized and stained for rNadA. The drugs were present during the entire incubation period. IF intensity was calculated as mean ± s.e.m in two independent experiments, each assessing 10–15 cells and expressed as Arbitrary Units (A.U.). Scale bar 10 µm.

The functional role of external Hsp90 in rNadA internalization was further explored by means of a synthetic membrane-impermeable inhibitor, the FITC-GA, that specifically target the extracellular population of the chaperone. To verify that FITC-GA did not enter the cells we used the cellular AKT levels as a marker for the functionality of the intracellular chaperone [66]. Hsp90 inhibitors (e.g. 17-AAG) promote the proteasomal degradation of this intracellular kinase. Cells were incubated with medium containing 2.5 and 10 µM of either 17-AAG or FITC-GA for 1, 4, 24 and 48 hrs. At the end of each period, cell lysates were prepared and equal amount of proteins were analyzed by electrophoresis and Western blot to reveal the amount of intracellular AKT. As shown in figure 8A, the levels of AKT did not change until 4 hrs of treatment with the inhibitors (figure 8A, lanes 2–7). At 24 and 48 hrs the intracellular kinase was degraded in cells incubated in presence of 17-AAG at both concentrations (figure 8A, lanes 9–10, 13–14) while its amount was not modified in FITC-GA treated cells (figure 8A, lanes 11–12, 15–16). This result strongly suggested that the Hsp90 inhibitor FITC-GA do not exert any activity on the intracellular population of the chaperone.

To verify that the membrane-impermeable inhibitor was an efficient Hsp90 inhibitor, we transiently permeabilized the cells. Chang cells were treated with saponin for 30 seconds in presence of FITC-GA and, once cell integrity was restored, incubated for 1, 4, 24 and 48 hrs. The amount of intracellular AKT was then detected as previously described. As shown in figure 8B, intracellular FITC-GA was revealed to be an efficient Hsp90 inhibitor leading to AKT degradation within 24 hours (figure 8B, lanes 11–18).

Chang cells were preincubated with either 10 µM 17-AAG or 10 µM FITC-GA at 37°C for 1 hr before addition of rNadA, and were further incubated for 1 and 4 hrs in presence of the inhibitors. Cells were then fixed, stained and subjected to confocal microscopy analysis (figure 8C). Compared to the control (upper panel of figure 8C), both inhibitors led to a similar accumulation of intracellular rNadA.

Taken together, the use of these inhibitors confirmed that the rNadA intracellular trafficking is somehow dependent upon functionally active Hsp90 and emphasized the role of the extracellular pool of this chaperone.

### Colocalization of rNadA with Rab11 and effect of inhibitors

**Figure 9:**
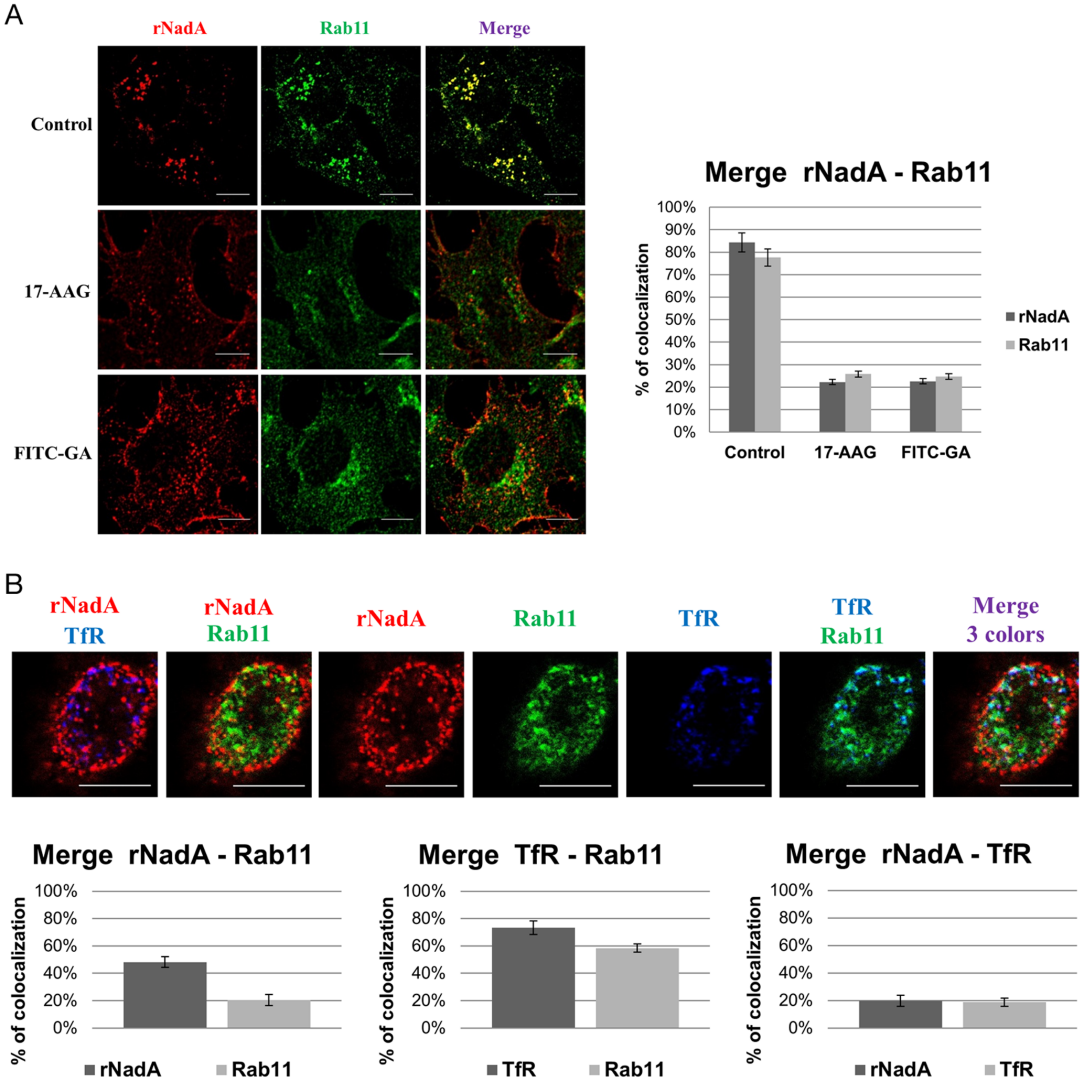
rNada internalization and colocalization with Rab11 and TfR. ***A: rNadA and Rab11 colocalization in presence of inhibitors***. The control untreated Chang cells are shown in the upper panel. Pre-treatment of cells was performed for 1 hour with 10 µM 17-AAG (middle panel) or 10 µM FITC-GA (bottom panel). Cells were then incubated with 200 µg/ml rNadA at 37°C for 1 hour. Afterward, cells were fixed, permeabilized and stained for NadA. The drugs were present during the entire incubation period. *Graph*: rNadA columns indicate the percentage of rNadA immunofluorescent pixels colocalizing with Rab11 immunofluorescent pixels in untreated, 17-AAG and FITC-GA cells (from left to right) respectively. Conversely, Rab11 columns indicate the percentage of Rab11 immunofluorescent pixels colocalizing with rNadA immunofluorescent pixels in untreated, 17-AAG and FITC-GA cells (from left to right) respectively. ***B: Rab11, rNadA and TfR colocalization*** **.** *A:* Chang cells were transfected with EGFP-Rab11 plasmid, incubated 24 hours at 37°C. and then incubated with 200 µg/ml of rNadA at 37°C for 2 hours. Afterward, cells were fixed, permeabilized and stained for NadA (Red) and TfR (blu). Rab11 is colored in green. *Graphs*: rNadA columns indicate the percentage of rNadA immunofluorescent pixels colocalizing with Rab11 fluorescent pixels (left) or with TfR immunofluorescent pixels (right). Rab11column indicate the percentage of fluorescent pixels colocalizing with rNadA immunofluorescent pixels (left) or TfR immunofluorescent pixels (right). TfR columns indicate the percentage of TfR immunofluorescent pixels colocalizing with Rab11 fluorescent pixels (middle) or with rNadA immunofluorescent pixels (left). Data are mean ± s.e.m representative of two independent experiments, each assessing 20–25 cells.

Our data strongly suggested that rNadA is reclycled back to the plasma membrane and possibly released back to the cell culture medium. Although several Rabs are known to regulate cellular membrane traffic [43], [67], Rab11 has been particularly associated with recycling endosomes and vesicular exocytosis at the plasma membrane [68]. We hypothesized that rNadA was colocalizing with Rab 11. Chang cells were incubated with rNadA for 1 hr and then stained for rNadA and Rab11. Confocal microscopy revealed that vesicles containing rNadA were also associated with Rab11 (figure 9A, upper panel) strongly suggesting that the adhesin moved into a recycling pathway.

We also investigated the Rab11-rNadA colocalization in presence of the two Hsp90 inhibitors. Chang cells were pre-treated with 17-AAG, FITC-GA for 1 hr before adding rNadA and allow internalization for one more hr at 37°C in presence of the inhibitors. Confocal microscopy analysis revealed a different cellular distribution of Rab11 in the presence of either drug (figure 9A, middle and lower panels). In the absence of inhibitors, Rab11 was mostly localized in vesicular structures (figure 9A, upper panel). In presence of 17-AAG or FITC-GA the Rab11 cellular pattern was more distributed, with a tendency to perinuclear accumulation (figure 9, middle and lower panels). Interestingly, both 17-AAG (membrane permeable Hsp90 inhibitor) and FITC-GA (membrane-impermeable inhibitor) treatment resulted in the complete loss of Rab11-rNadA colocalization (figure 9, compare right panels) whereas intracellular accumulation of rNadA was observed. Treatment with the Hsp90 inhibitors did not interfere with the co-localization of TfR with Rab11 positive endosomes (figure S3).

Taken together, these observations indicated that Hsp90 activity was not essential for the formation of vesicles containing rNadA. Rather, it may affect the recruitment of effector molecules necessary for the formation of recycling endosomes.

Due to the high level of colocalization between internalized rNadA and Rab11, we reasoned that it was surprising not to have found rNadA-TfR colocalization in our previous experiment (figure 4E–F). Indeed, TfR recycling, has been reported to rely on Rab11 endosomes [43]. To decipher this apparent contradiction, we performed a simultaneous staining of TfR and Rab11 during rNadA internalization. Chang cells were transfected with an EGFP-Rab11 expressing plasmid and 24 hours later incubated with rNadA for 2 hours. Cells were then fixed and revealed for rNadA and TfR by using specific primary antibodies. Results from confocal microscopy analysis are shown in figure 9B in which, to better discriminate colocalization between pair of proteins, two colors at the time are also shown separately. Furthermore, percentages of colocalization are reported in the graphs of figure 9B.

Confocal microscopy revealed a significant colocalization of rNadA in Rab11 vesicles (figure 9B, upper panel and left graph) although the value was lower than previous experiment. This could be due to the different method used to reveal Rab11 in the experiments of figure 9A and 9B. Whereas physiological levels of Rab11 were detected in figure 9A by specific antibody, overexpression of a fluorescent tagged protein increase the amount of endogenous GTPase and may justify the lower percentage of association. Colocalization of TfR with Rab11 was significantly higher, consistent with data from the literature (figure 9A upper panel and middle graph), but rNadA and TfR did not show contemporary presence in Rab11 vesicles (figure 9B upper panel and right graph).

These data suggest that the rNadA and TfR enter distinct population of Rab11 endosomes. Although surprising, the existence of separate sub-population of Rab11 vesicles has already been observed in a different cell type [69].

## Discussion

In this report we have shown that a purified bacterial adhesin binds human epithelial cells and triggers its own internalization. Once internalized, the intracellular trafficking of rNadA avoids lysosome targeting by entering an endosome recycling pathway and is likely transported back to the extracellular space. The extracellular pool of the molecular chaperone Hsp90, a cellular chaperone previously shown to bind rNadA *in vitro* [30], has been identified as a crucial factor for the intracellular trafficking of the meningococcal adhesin.

The early steps of rNadA endocytosis remain poorly understood and, in particular, we still have not defined the cellular ligand on the membrane. Binding of rNadA to Chang cells is strongly temperature dependent and it dramatically drops below 30°C. Internalization of the adhesin is a slow event (about 30 min) compared to general mechanisms of endocytosis. In the last decade, the endocytic process has shown a surprising diversity of parallel mechanisms in a single cell [38], [70]. The rNadA entry process in human epithelial cells is clathrin-independent, insensitive to general tyrosine kinases or specific Src inhibitors, and thus excludes the involvement of a caveolin dependent entry pathway [38]. Treatment with the PI3K inhibitor wortmannin, in contrast, led to rNadA accumulation at the cell periphery, consistent with the role played by this kinase in the early steps of vesicle formation [71], [72]. Endocytic vesicles containing rNadA enter an ARF6-regulated trafficking pathway and over-expression of a constitutively activated form of this protein (Q67L) led to the rNadA accumulation in vacuolar structures. Activated ARF6 is described to have a dual role in cells: it promotes internalization at the level of the plasma membrane and is a targeting factor for recycling of cargo to the cell surface [43], [73]. Our data suggested that rNadA could avoid the lysosomes by steering into a recycling route. Thus far, a limited number of proteins have been described to use an ARF6-regulated pathway [43], [63], the list including β1-integrin and MHC-I that are continuously trafficking from endocytic vesicles back to the plasma membrane. Consistent with our results, expression of ARF6 Q67L mutant has been shown to induce vacuolar structures and blocks recycling to the cell surface of both β1-integrin and MHC-I [74]. The hypothesis that rNadA enters a recycling pathway was further reinforced by its significant colocalization with Rab11 coated vesicles. The small GTPase Rab11 is a recognized marker of recycling endosomes, and a crucial factor regulating the exocytic fusion event [43], [67], [68], [75]. Interestingly, stimulation-dependent recycling of β1-integrin follows an Arf-6 and Rab11 dependent pathway [76]. This integrin has been recently indicated as a putative ligand for NadA by using a heterologous system of invasion [77]. In our hands, the anti-β1 antibody failed to impair both binding and internalization of rNadA in Chang cells (GB and MM unpublished results) but, due to the different experimental system used, a role for this integrin in NadA-mediated binding and/or internalization cannot be ruled out. Intriguingly, we found that Rab11 positive endosomes containing rNadA are distinct from Rab11 positive endosomes carrying TfR, although formation of both populations is PI3K dependent. Although we have not further characterized the identity of these vesicles, the existence of diversified Rab11 endosomes has been recently reported in the rat adrenal phaeochromocytoma cell line PC12 [69].

Our attempts to identify a rNadA cell surface ligand led us to extracellular Hsp90 [30], a finding that was independently supported by another laboratory [53]. In the present paper, we have shown that external Hsp90 internalizes and colocalizes with the rNadA containing endocytic vesicles in live cells, strongly suggesting that the initial interaction between rNadA and Hsp90 takes place in the extracellular milieu. The extracellular presence of Hsp90 has been controversial for a long time, but it is now accepted that secretion of this chaperone occurs in both tumor and normal cells. Moreover, the functions of extracellular Hsp90 do not rely on the classical chaperone ATPase activity [49], [51]. In our previous study we demonstrated a direct binding *in vitro* between rNadA and a complex of Hsp90 and ADP or 17-AAG. Meanwhile, in the presence of ATP, no such binding was observed [30]. Although the internalization of rNadA is not impaired by interfering with Hsp90, the ensuing intracellular trafficking is affected. Here we show that rNadA uptake in presence of the membrane-impermeable Hsp90 inhibitor FITC-GA led to intracellular accumulation of vesicles that contained the adhesin but were unable to engage Rab11. Interaction of rNadA with external Hsp90, thus, would be required to recruit a pattern of effector molecules for the targeting of the adhesin to the recycling endosomes. On the other hand, involvement of Hsp90 activity in intracellular trafficking has been poorly documented in literature. Its activity is required for activation of the Rho family of small G-protein that are known to regulate cytoskeleton rearrangement and endosomal trafficking [78], [79]. Very recently, Cortese et al. reported that recycling of ErbB2 is perturbed by cell treatment with the Hsp90 inhibitor geldanamycin, leading to targeting of the receptor to mixed endosomal compartments [80]. To our knowledge, however, there are no reports on the influence of external Hsp90 on intracellular trafficking. The data presented in this work led us to hypothesize that the binding of rNadA to extracellular Hsp90 may influence a selection of the signaling involved in internalization, but further studies are required to elucidate this aspect.

The role of adhesins in meningococcal pathogenesis is thought to be confined to securing the bacteria to the host cell surface, whereas their contribution to bacterial trafficking is unaddressed [4]. We believe that the model exploited in this work, through the dissection of the pathways employed by an individual adhesin, has shed new light on the possible role that “minor” adhesion factors may have in the pathogenesis of bacterial invasion and, in the context of *N. meningitidis*, in transcellular passage through the epithelial barrier.

## Supporting Information

Figure S1 **Size Exclusion - HPLC coupled with a MALLS (Multi Angle Laser Light Scattering) instrument of rNadA heated at 37°C for different times.** ***Panel A:*** SE-HPLC profile of rNadA at room temperature. ***Panel B, C,D, E:*** SE-HPLC profiles of rNadA after heating period at 37°C of 30 min, 1, 2 and 4 hrs respectively. ***Panel F:*** Comparison of the different SE-HPLC profiles of rNadA heated at 37°C for the indicated times. In the panel A, the diverse species eluted at different elution time can be unequivocally identified having been previously characterized [33]. Equilibrated at room temperature, rNadA preparation showed a predominant molecular weight corresponding to the native trimer (peak II) and three minor peaks corresponding to aggregates (peak I of figure 2A), monomer (peak III of figure 2A) and C-deleted monomer (peak IV of figure 2A). The NadA preparation, includes a certain number of C-deleted forms that are able to form trimers and are eluted in the peak II [33]. Incubation at 37°C did not provoke any difference in the SE-HPLC retention time and the MALLS measured MW of NadA peaks revealed at room temperature but it induced changes in their relative percentages.
(TIF)

Figure S2 **Colocalization of Transferrin receptor with clathrin.** Chang cells were fixed, permeabilized and double stained for clathrin (red) and Transferrin receptor (green). Merged image is also shown.
(TIF)

Figure S3 **Colocalization of Transferrin receptor with Rab11 in presence of Hsp90 inhibitors.** The control untreated Chang cells are shown in the upper panel. Pre-treatment of cells was performed for 1 hour with 10 µM 17-AAG (middle panel) or 10 µM FITC-GA (bottom panel). Chang cells were then fixed, permeabilized and double stained for Rab11 (green) and transferrin receptor (blu). Merged images are also shown. Graph report the percentage of colocalization obtained by 3 independent experiments.
(TIF)

Table S1 **Relative percentages on rNadA species heated at 37°C for different times as revealed by Size Exclusion - HPLC coupled with a MALLS (Multi Angle Laser Light Scattering) in figure S1.**
(TIF)

Movie S1 **Dynamics of rNadA and MHC-I internalization in live cells.** Chang cells were incubated for 1 h with Alexa488-conjugated anti-MHC-I antibody (green) and Alexa633-conjugated rNadA (red). Cells were then washed and imaged at the confocal microscope. Images were taken every 2 seconds.
(AVI)

Movie S2 **Dynamics of rNadA and transferrin internalization in live cells.** Chang cells grown overnight were incubated for 1 h with Alexa488-conjugated rNadA (green) and cy3-conjugated transferrin (red). Cells were then washed and imaged at the confocal microscope. Images were taken every 4 seconds.
(AVI)

Movie S3 **Dynamics of rNadA in 17-AAG treated cells.** Chang cells, pre-treated overnight with 0.5 µM 17-AAG (17-AAG), were incubated for 1 h with Alexa488-conjugated rNadA (green), in presence of the the drug. Cells were then washed and imaged at the confocal microscope. Images were taken every 4 seconds.
(AVI)

Movie S4 **Dynamics of rNadA in untreated cells.** Chang cells, pre-treated overnight with vehicle, were incubated for 1 h with Alexa488-conjugated rNadA (green). Cells were then washed and imaged at the confocal microscope. Images were taken every 4 seconds.
(AVI)
