# Supplementary figures and images for: Role of ARF6, Rab11 and External Hsp90 in the Trafficking and Recycling of Recombinant-Soluble Neisseria meningitidis Adhesin A (rNadA) in Human Epithelial Cells
Source: PLoS One. 2014 Oct 27;9(10):e110047. doi: 10.1371/journal.pone.0110047 (PMC4210143; doi:10.1371/journal.pone.0110047)

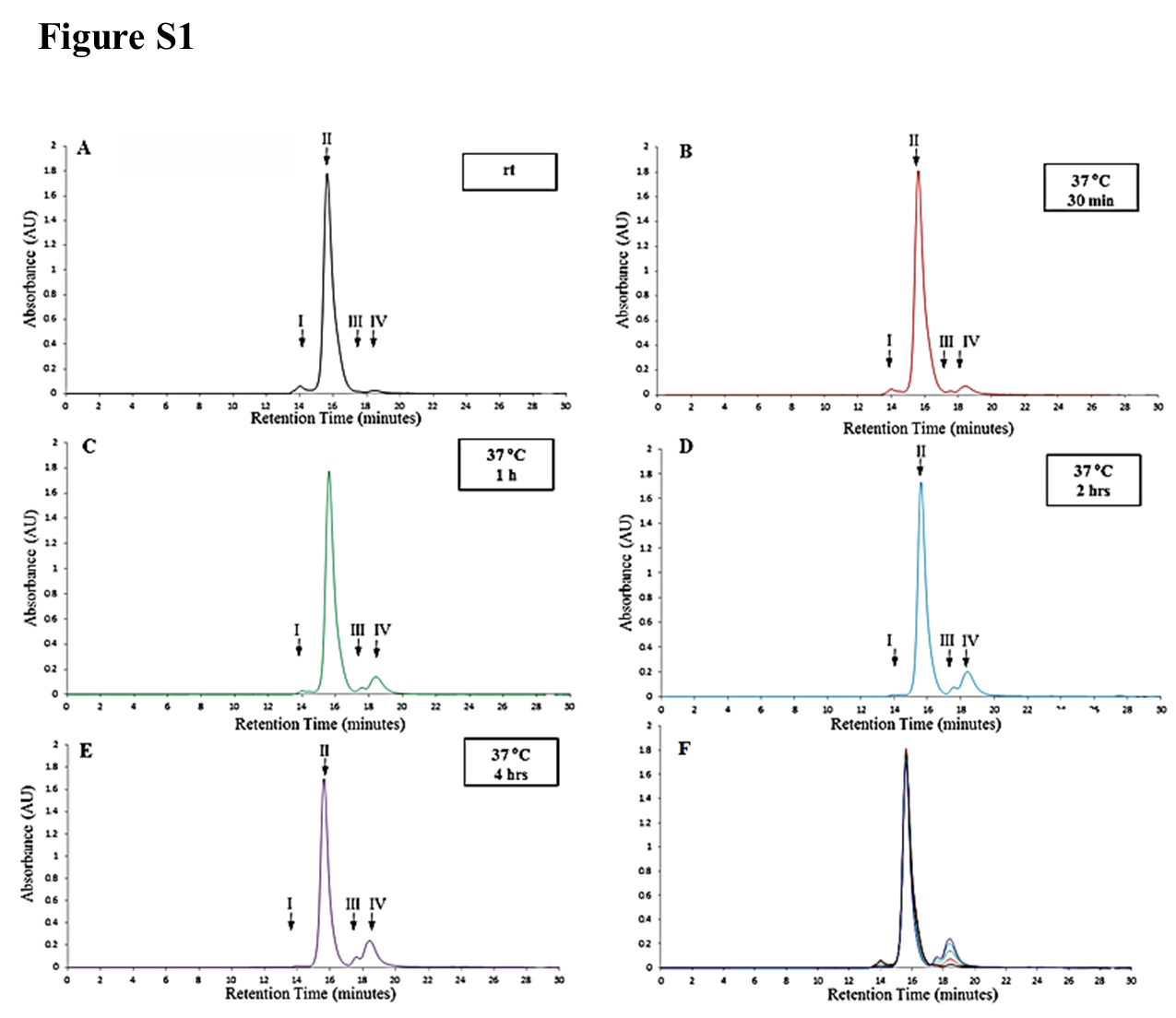

Supplement: Figure S1 — Size Exclusion - HPLC coupled with a MALLS (Multi Angle Laser Light Scattering) instrument of rNadA heated at 37°C for different times. Panel A: SE-HPLC profile of rNadA at room temperature. Panel B, C,D, E: SE-HPLC profiles of rNadA after heating period at 37°C of 30 min, 1, 2 and 4 hrs respectively. Panel F: Comparison of the different SE-HPLC profiles of rNadA heated at 37°C for the indicated times. In the panel A, the diverse species eluted at different elution time can be unequivocally identified having been previously characterized [33]. Equilibrated at room temperature, rNadA preparation showed a predominant molecular weight corresponding to the native trimer (peak II) and three minor peaks corresponding to aggregates (peak I of figure 2A), monomer (peak III of figure 2A) and C-deleted monomer (peak IV of figure 2A). The NadA preparation, includes a certain number of C-deleted forms that are able to form trimers and are eluted in the peak II [33]. Incubation at 37°C did not provoke any difference in the SE-HPLC retention time and the MALLS measured MW of NadA peaks revealed at room temperature but it induced changes in their relative percentages. (TIF) [file pone.0110047.s001.tif]

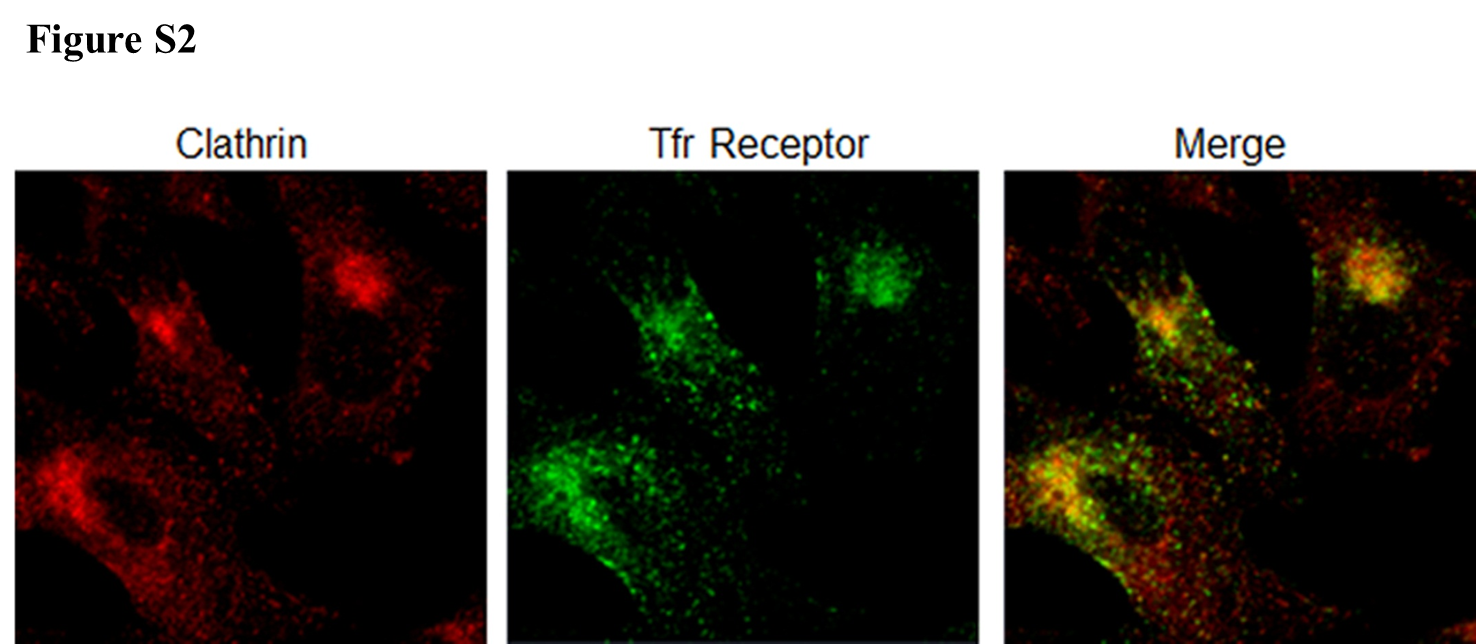

Supplement: Figure S2 — Colocalization of Transferrin receptor with clathrin. Chang cells were fixed, permeabilized and double stained for clathrin (red) and Transferrin receptor (green). Merged image is also shown. (TIF) [file pone.0110047.s002.tif]

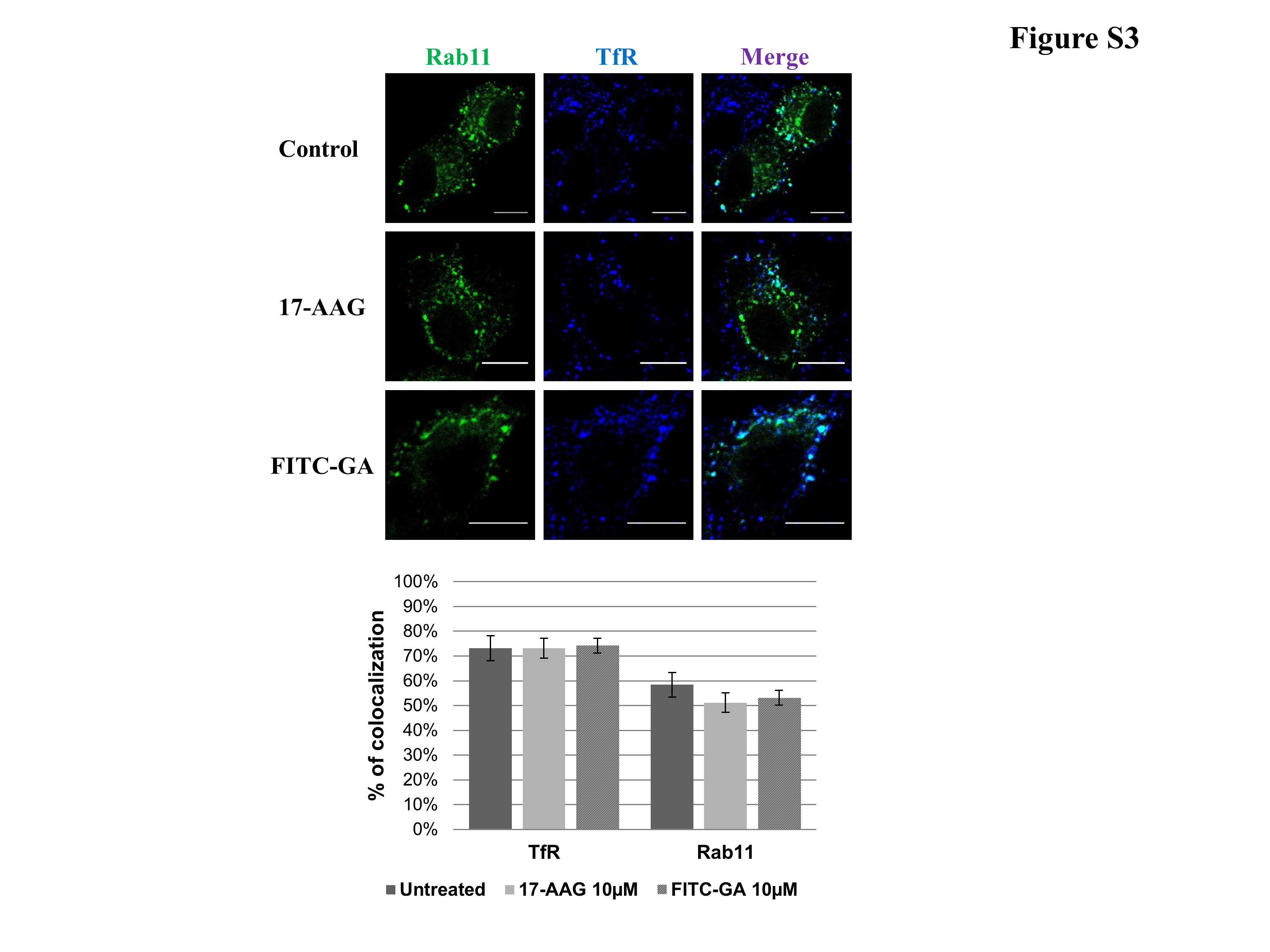

Supplement: Figure S3 — Colocalization of Transferrin receptor with Rab11 in presence of Hsp90 inhibitors. The control untreated Chang cells are shown in the upper panel. Pre-treatment of cells was performed for 1 hour with 10 µM 17-AAG (middle panel) or 10 µM FITC-GA (bottom panel). Chang cells were then fixed, permeabilized and double stained for Rab11 (green) and transferrin receptor (blu). Merged images are also shown. Graph report the percentage of colocalization obtained by 3 independent experiments. (TIF) [file pone.0110047.s003.tif]

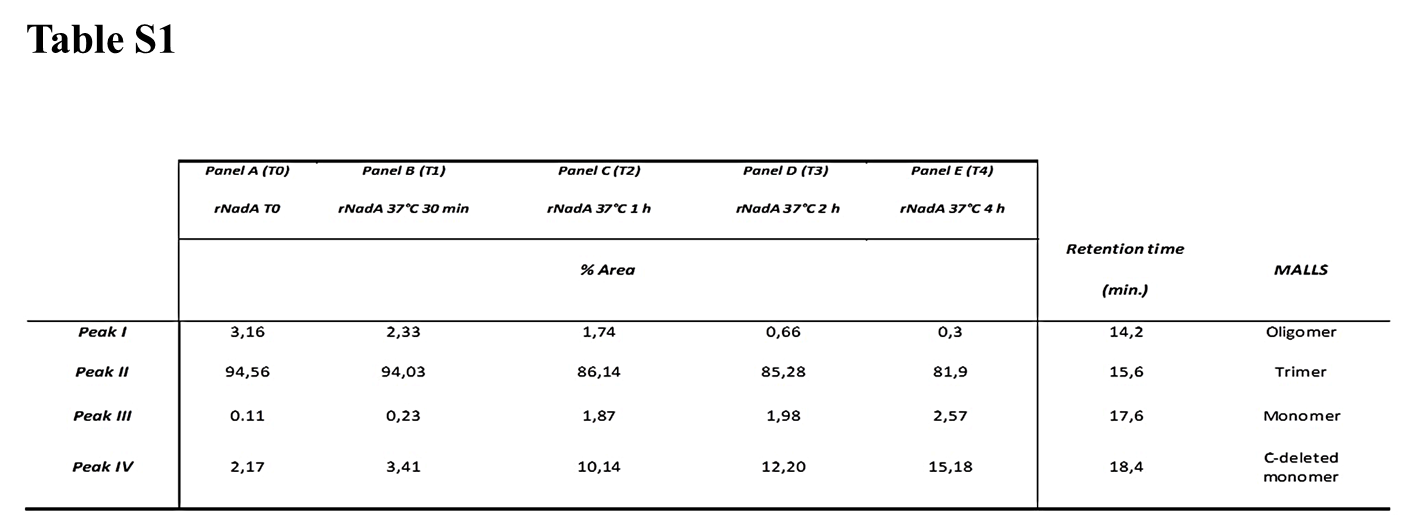

Supplement: Table S1 — Relative percentages on rNadA species heated at 37°C for different times as revealed by Size Exclusion - HPLC coupled with a MALLS (Multi Angle Laser Light Scattering) in figure S1. (TIF) [file pone.0110047.s004.tif]
